# Telephone triage utilization among patients with limited English proficiency

**DOI:** 10.1186/s12913-017-2651-z

**Published:** 2017-11-09

**Authors:** Jane W. Njeru, Swathi Damodaran, Frederick North, Debra J. Jacobson, Patrick M. Wilson, Jennifer L. St Sauver, Carmen Radecki Breitkopf, Mark L. Wieland

**Affiliations:** 1 Division of Primary Care Internal Medicine, Department of Medicine, Mayo Clinic, 200 First Street SW, Rochester, MN 55905 USA; 2 Mayo Medical School, Mayo Clinic College of Medicine, Rochester, MN USA; 3 Division of Biomedical Statistics and Informatics, Department of Health Sciences Research, Rochester, MN USA; 4 Robert D and Patricia E Kern Center for the Science of Health Care Delivery, Rochester, MN USA; 5 Department of Health Sciences Research, Mayo Clinic, Rochester, MN USA

**Keywords:** Telephone triage, Limited English proficiency, Interpreter services, Immigrants and refugees

## Abstract

**Background:**

Communication between patients with limited English proficiency (LEP) and telephone triage services has not been previously explored. The purpose of this study was to determine the utilization characteristics of a primary care triage call center by patients with LEP.

**Methods:**

This was a retrospective cohort study of the utilization of a computer-aided, nurse-led telephone triage system by English proficiency status of patients empaneled to a large primary care practice network in the Midwest United States. Interpreter Services (IS) need was used as a proxy for LEP.

**Results:**

Call volumes between the 587 adult patients with LEP and an age-frequency matched cohort of English-Proficient (EP) patients were similar. Calls from patients with LEP were longer and more often made by a surrogate. Patients with LEP received recommendations for higher acuity care more frequently (49.4% versus 39.0%; *P* < 0.0004), and disagreed with recommendations more frequently (30.1% versus 20.9%; *P* = 0.0004). These associations remained after adjustment for comorbidities. Patients with LEP were also less likely to follow recommendations (60.9% versus 69.4%; *P* = 0.0029), even after adjusting for confounders (adjusted odds ratio [AOR] = 0.65; 95% confidence interval [CI], 0.49, 0.85; *P* < 0.001).

**Conclusion:**

Patients with LEP who utilized a computer-aided, nurse-led telephone triage system were more likely to receive recommendations for higher acuity care compared to EP patients. They were also less likely to agree with, or follow, recommendations given. Additional research is needed to better understand how telephone triage can better serve patients with LEP.

## Background

Systems for telephonic triage of health concerns have become increasingly common as a cost-effective means of improving access to medical advice and care [1]. In the primary care setting, these triage call centers are typically staffed by nurses who use computer- assisted decision-making tools to provide care recommendations to callers and manage patient consultation requests [2]. This consultation management has been shown to effectively manage patient access to providers while reducing workload and maintaining costs [3–6]. In the broader healthcare context telephonic triage systems may reduce costs by directing patients to less resource intensive encounters (e.g., primary care appointment instead of an emergency department (ED) visit) [7, 8]. Computer-assisted triage call centers have also been shown to maintain safety standards in providing medical recommendations over the telephone and are effective in assessing the appropriateness of care decisions [9, 10]. Questions still remain, however, about patient adherence rates with nurse recommendations [11], which may be affected by the quality of communication between the patient and the triage nurse [12]. For instance, some callers may misinterpret nurse recommendations while others choose not to adhere to recommendations due to their own intentions, health beliefs, or social circumstances [12, 13].

Quality of healthcare communication is a particularly salient issue among patients with limited English proficiency (LEP). LEP is defined as “speaking English less than very well” in any person 5 years of age and older, and it is associated with disparities in healthcare utilization and health outcomes [14, 15]. Telecommunication studies examining interactions between healthcare systems and patients with LEP have demonstrated barriers and negative outcomes related to emergency medical service calls [16, 17]. Disparities in utilization of telephone triage have also been demonstrated among non-native language speakers in countries outside the U.S. Non-native Swedish speakers utilized a national phone triage service less frequently compared to native speakers, while non-native Norwegian speakers reported less trust in recommendation/advise given to them by nurses through telephone triage [18, 19]. To help mitigate these barriers, healthcare systems typically contract with professional medical interpreters and telephonic or video interpretation services. Interpreter use reduces, but does not eliminate health disparities among patients with LEP, partly due to the type of interpreter used and the quality of interpretation services [20, 21].

Communication between patients with LEP and primary care telephone triage services which use computer-assisted decision making algorithms has not been previously examined. As the use of triage call centers becomes more prevalent and the proportion of patients with LEP continues to grow, it is important to understand utilization patterns within these populations. Moreover, given that significant disparities in care exist within face-to-face healthcare encounters/systems, the potential exists for exacerbating disparities when communication is by phone. Therefore, the purpose of this study was to determine the utilization of a primary care triage call center by patients who require interpreter services.

## Methods

This was a retrospective cohort study of the utilization characteristics and outcomes of computer-aided nurse telephone triage calls by English proficiency status of patients empaneled to a large primary care practice network in the Midwest United States between 1/1/2012 and 6/30/2013. Patient data were accessed only if they had an active Minnesota research authorization on file, allowing use of their medical records for research, [22] and all study procedures were approved by the Mayo Clinic Institutional Review Board.

### Nurse triage protocol

All patients empaneled to this academic primary care practice have access to telephone triage. When patients or their surrogates call the clinic for assessment of symptoms, their calls are routed to the experienced nurses who staff the triage call center, and using ExpertRN, a Mayo Clinic proprietary computer-assisted triage decision support system available 24 h a day, every day, they advise patients on the next steps in the management of their symptoms. There were 67,494 calls made by 35,139 unique practice empaneled patient callers in 2013.

### Participants

Adult patients (≥18 years of age) were eligible for inclusion if they were actively empaneled to receive primary care in the Internal Medicine or Family Medicine Departments in the healthcare network. To identify the subset of patients with LEP, patient-identified need for interpreter services (IS) was used as a proxy. Patient IS status is readily and accurately available in administrative datasets through electronic medical records, and has been used as an indirect measure of LEP in previous studies [23]. The cohort of non-IS (English proficient) patients was derived through age-frequency matching to the IS cohort.

### Measures

Patient registration and billing data were used to obtain the following for each patient: age; gender; ethnicity/race; interpreter status; and outpatient healthcare utilization defined as the number of office visits to primary care clinics during the study interval. Medical complexity was calculated by defining the Charlson Comorbidity Index (CCI) for each patient. The CCI is a method used to classify and weight comorbid conditions as a means of measuring disease burden and predicting mortality [24].

The institutional primary care telephone triage database was used to obtain the following data for each patient: total number of calls during the study interval; call characteristics (chief symptom for call, mean duration of call); caller type (self or surrogate); and triage recommendations for the call (home care, provider advice, routine visit within 3 days, acute appointment within 24 h, acute appointment in 4 h, or emergency visit).

Following each triage phone call a unique document is created in the patient’s electronic health record that, in addition to the call characteristics, contains detailed information on symptoms, whether the caller agrees with the recommendations given by the nurse, and the caller’s intention to complete or not complete the recommended action(s). A single author (S.D.) performed a manual chart review of the electronic health record of every patient for the 4 weeks following the call to assess whether the patient followed the call recommendation or not. A second author (J.W.N.) performed random checks of 20% of the chart reviews to ensure accuracy and decrease chances of bias. If there was more than one call during the study interval, the first call was used to record this measure. Our analysis was by unique caller and not by call. Since many of the 587 patients in both the IS and non-IS groups called multiple times during the study interval, a decision was made to use the first call during the study interval for our analysis.

### Data analysis

Patient demographics and call characteristics were compared by IS status using a chi-square test for categorical variables and Wilcoxon rank sum test for continuous variables. Number of phone calls per person was categorized as 1, 2, and 3 or more. Logistic regression was used to assess the association between IS status with adherence to the call recommendation. Multivariable models were used to adjust for potential confounding effects of gender, CCI score, call duration, person who placed the call (surrogate or self), and recommended action. Interactions were assessed and those found to be significant were presented using stratified models to compare the association of IS status with call follow through by recommended action and by caller (surrogate or self).

## Results

### Caller characteristics

The study cohort included 587 IS callers and an age-frequency matched cohort of 587 non-IS callers (Table 1).

**Table 1:** Demographic Characteristics of IS Call Patients and Age-Frequency Matched Non-IS Patients

	Non-IS (*N* = 587)	IS (N = 587)	*P* value^1^
Age (in years), n (%)			1.0000
18–30	74 (12.6)	74 (12.6)	
31–40	109 (18.6)	109 (18.6)	
41–50	81 (13.8)	81 (13.8)	
51–60	109 (18.6)	109 (18.6)	
61–70	104 (17.7)	104 (17.7)	
> 70	110 (18.7)	110 (18.7)	
Gender, n (%)			0.1186
Female	385 (65.6)	410 (69.8)	
Male	202 (34.4)	177 (30.2)	
Language^2^			<0.0001^1^
Arabic	3 (0.5%)	66 (11.2%)	
Asian	20 (3.4%)	193 (32.9%)	
English	560 (95.4%)	0 (0.0%)	
Somali	3 (0.5%)	225 (38.3%)	
Spanish	1 (0.2%)	39 (6.6%)	
Other	0 (0.0%)	64 (10.9%)	
Charlson score, n (%)			0.0183
0	207 (35.3)	182 (31.0)	
1	169 (28.8)	144 (24.5)	
2	70 (11.9)	99 (16.9)	
≥ 3	141 (24.0)	162 (27.6)	

*Abbreviations*: *IS* Interpreter Services

^1^ *P*-value from Chi-square test

^2^Language: 4.6% of the non-IS patients had a language other than listed English as their primary language, but did not require an interpreter

The median (Q1, Q3) age of callers was 53 (36, 67) years and callers were more frequently female in both groups. IS callers had higher CCI scores compared to non-IS callers (*P* = 0.0183). Among the IS callers, the most common languages were Somali (38.3%), Asian languages -mainly Vietnamese, Cambodian and Mandarin (32.9%) and Arabic (11.2%).

### Call characteristics and nurse recommendation

Compared to calls from non-IS patients, those from IS patients were of longer duration in minutes; Median (Q1, Q2): 13.9 (9.2, 21.1) versus 12.2 (7.9, 18.2); *P* = 0.0002, and were more often made by a surrogate, n (%): 203 (34.6%) versus 35 (6.0%); *P* < 0.0001 (Table 2).

**Table 2:** Call Characteristics and Outcomes of IS Call Patients and Age-Frequency Matched Non-IS Patients

	Non-IS (N = 587)	IS (N = 587)	*P* value
Duration of call (minutes)			0.0002^1^
Median (Q1,Q2)	12.2 (7.9, 18.2)	13.9 (9.2, 21.1)	
Person who placed call, n (%)			<0.0001^2^
Surrogate	35 (6.0)	203 (34.6)	
Self	552 (94.0)	384 (65.4)	
Number of calls, n (%)			0.2426^2^
1	315 (53.7)	331 (56.4)	
2	144 (24.5)	151 (25.7)	
3+	128 (21.8)	105 (17.9)	
Recommended action,^3^ n (%)			0.0004^2^
Advice/Home Care/Treatment within 24 h	234 (40.6)	187 (33.5)	
Ambulance/ED visit now	56 (9.7)	96 (17.2)	
Routine visit within a week	117 (20.3)	95 (17.0)	
Urgent visit/Acute appointment	169 (29.3)	180 (32.2)	
Caller agrees with recommendation,^3^ n (%)			0.0004^2^
Yes	457 (79.1)	380 (69.9)	
No	121 (20.9)	164 (30.1)	
Recommendation followed,^3^ n (%)			0.0029^2^
Yes	379 (69.4)	339 (60.9)	
No	167 (30.6)	218 (39.1)	

*Abbreviations*: *ED* Emergency Department, *IS* Interpreter Services

^1^ *P*-value from Wilcoxon test

^2^ *P*-value from Chi-square test ^ 3^N and percent based on non-missing values

There was no significant difference in the number of calls placed between the two groups. Nurse recommendations for higher acuity care, (call an ambulance, visit the ED, or schedule an acute appointment) were more frequent for IS callers than non-IS callers (49.4% versus 39.0%; *P* < 0.0004), while non-IS callers received recommendations for less acute care (home care and a routine visit within 1 week) more frequently than IS patients (60.9% versus 50.5%; *P* < 0.0001). These differences remained significant after adjustment for comorbidities (data not shown). There were no significant differences in the chief symptom for calls between the two groups.

### Caller agreement with nurse recommendation

The IS callers were less likely to agree with the recommendations given by the nurse, compared to the non-IS callers (n (%) 164 (30.1%) versus 121 (20.9%); *P* = 0.0004) (Table 2). This association remained significant after adjustment for comorbidities (data not shown).

### Caller adherence to nurse recommendation

The IS patients were also less likely to follow the recommendations given by the nurse, n (%): 339 (60.9%) versus 379 (69.4%); *P* = 0.0029. After adjusting for sex, CCI, caller type (self or surrogate), duration of call, and recommended action, IS callers were less likely to follow the nurse’s recommendation than non-IS callers (adjusted odds ratio [AOR] = 0.65; 95% confidence interval [CI], 0.49, 0.85; *P* < 0.001) (Table 3). When stratified by recommended action (*P* for interaction <0.0001), IS patients were less likely to follow through with recommendations to call an ambulance or visit the ED (AOR = 0.28; 95% CI, 0.13, 0.60) and recommended home care (AOR = 0.34; 95% CI, 0.22, 0.55), but were more likely for follow through with the recommendation for a routine visit within a week (AOR = 2.45; 95% CI, 1.24, 4.82; Table 3). When stratified by person calling (*P* for interaction =0.019), IS patients who used a surrogate were less likely to follow through with the nurse recommendation compared to non-IS patients (AOR = 0.21; 95% CI, 0.07, 0.65) than IS patients who called for themselves (AOR = 0.75; 95% CI, 0.51, 1.00).

**Table 3:** Association of IS Status with the Patient Following Through with Recommendation, Overall and Stratified by Recommended Action and Person Calling

Overall	Unadjusted OR (95% CI)	Adjusted OR (95% CI)
Non-IS	1.0	1.0^a^
IS	0.65 (0.51, 0.84)	0.65 (0.49, 0.85)
Recommended Action^c^		
Advice/Home Care/Treatment within 24 h	0.37 (0.24, 0.57)	0.34^b^ (0.22, 0.55)
Routine visit within a week	2.45 (1.27, 4.70)	2.45 (1.24, 4.82)
Urgent visit/Acute appointment	0.90 (0.57, 1.42)	0.93 (0.59, 1.48)
Ambulance/ER visit now	0.29 (0.14, 0.60)	0.28 (0.13. 0.60)
Person Calling^c^		
Patient	0.76 (0.57, 1.00)	0.75^b^ (0.57, 1.00)
Surrogate for Patient	0.21 (0.07, 0.62)	0.21 (0.07, 0.65)

*Abbreviations*: *CI* Confidence Interval, *IS* Interpreter Services, *OR* Odds Ratio

^a^Models adjusted for sex, Charlson score, call duration, recommended action and person calling

^b^Models adjusted for sex, Charlson score and call duration

^c^ORs for following through with call recommendation for IS patients compared to non-IS patients stratified by recommended action and person calling

## Discussion

In this study of a computer-aided nurse-led telephone triage system in a primary care network, we found similar proportions of callers making one or repeat calls between patients who required IS and those who did not. However IS patients were more likely to receive recommendations for higher acuity care and urgent visits compared to non-IS patients. Furthermore, IS patients were much less likely to follow the recommendations received. To our knowledge, this study is the first to describe call characteristics and adherence to triage recommendations among patients with LEP. These results highlight the need for health systems to examine why LEP calls result in significantly lower adherence to triage recommendations and whether this lower adherence results in poor health outcomes or, perhaps, reflects a need to refine the triage process for patients with LEP.

Previous studies have shown patterns of inefficient healthcare utilization among patients with LEP compared with patients who are English-proficient. These studies reveal higher rates of use for diagnostic studies and increased ED visit times for patients with LEP, higher rates of outpatient and inpatient health care utilization [25–27], and longer hospital stays [28]. The results of this study support these previous findings, and suggest that these inefficiencies may be mediated, in part, by the tendency of primary care systems to disproportionately recommend higher acuity services to patients with LEP.

The reasons for the difference in recommendations given between the IS and non-IS groups that were demonstrated in this study are likely multifactorial. Although there were no significant differences in the chief symptom for calls between the two groups, we were unable to determine the severity of symptom complexes for each call; since IS patients were slightly more medically complex, it is possible that severity contributed to recommendations for more acute and aggressive care among the IS cohort. However, our findings did not change after adjusting for potential confounders, including CCI. Furthermore, previous studies have reported significant differences in care recommendations and patterns for patients with LEP despite similarities in presentation with English-proficient patients. For example, patients with LEP who presented to the ED with abdominal pain received three times as many abdominal computed tomographic scans as patients with English proficiency, while infants of parents with LEP were more likely to be prescribed antibiotics compared to those with parents proficient in English [29, 30]. One study of pediatric patients showed higher rates of hospital admission among patients with LEP compared to patients with English proficiency, although the acuity level at presentation was similar [31]. Communication may play the primary role as the mediator of the differences observed in the care recommendations between the two groups. The decision to recommend higher acuity of care may be influenced by a need on the part of the nurse to compensate for communication barriers by recommending more aggressive therapy options. This may be even more compelling in the case of telephone communication, where the healthcare provider does not have the benefit of examining the patient and where the patient may be communicating through a surrogate.

Our finding that patients with LEP were less likely to follow recommendations may reflect a disproportionate lack of understanding of those recommendations and their rationale among patients with LEP. The communication challenges of linguistic and cultural non-congruence between patients and nurses may be compounded by a relatively low health literacy, which is often interrelated with LEP, and has been independently associated with healthcare utilization and higher medical costs. [32–35] In this particular group of patients with LEP, the influence of health-seeking norms specific to their countries of origin may also play a significant role in their reduced acceptance of phone triage recommendations. For instance, in some recent immigrant groups, patients are only familiar with acute care models, which may prompt delays in seeking care until the perceived acuity escalates [36].

In this study, IS patients showed a higher rate of using a surrogate for triage calls compared to non-IS patients. Most surrogates are family members who serve as ad-hoc interpreters with no professional training. Several studies have examined the use of ad-hoc interpreters in healthcare settings and found an increased risk for poorer health outcomes due to interpretation errors and to family members imposing their own agendas on the care plan. [37, 38] The use of surrogates to make triage calls in the general population has also been shown to increase utilization rates for higher acuity services possibly due to surrogates anticipating these endpoints or exhibiting a higher level of concern for the patient’s symptoms. [39, 40] These findings in the general population may be potentiated among patients with LEP due to the added language barrier. While well-trained and qualified interpreters are the recommended standard of care among patients with LEP, further research is needed to explore the use of surrogates for triage calls in this population in order to better understand how their use affects outcomes and healthcare utilization.

Interestingly, our findings that IS patients were less likely to go to the ED when instructed but more likely to choose a routine appointment within 1 week suggests that IS patients may be choosing continuity with their primary care provider (or care team) over emergency or same day services with less familiar teams. This finding is encouraging given that previous research has found higher ED utilization among patients with LEP for dental, eye, skin, and ear, nose and throat concerns; most of which can be typically addressed in the outpatient setting [26]. Furthermore, encouraging patients with LEP to utilize primary care services for acute care needs may help address the existing inequities documented for management of chronic diseases among patients with LEP such as mental health, asthma, and diabetes mellitus [41–43]. However, it is also possible that this finding of less likelihood of visiting the ED when advised to may be because of fear of the financial implications of the ED visit and/or the ambulance transportation cost on the part of the caller.

Our study has several limitations. First, the data were collected from a single primary care triage call center, which limits the generalizability of our results. Furthermore, the demographics of patients with LEP in this sample may be different from other primary care centers serving patients with LEP. Second, the data collected was based on the assumption that those callers, who identified as needing IS in the electronic health record, were patients with LEP. Using IS as a proxy recognizes only a subset of patients with LEP [23]. Third, the study incorporated retrospective data that may be influenced by unidentified confounding factors or may be skewed due to missing data, such as patients seeking care in clinical sites other than the primary care practice where this study was done. However, measurable confounding factors were controlled for and there were minimal missing data. Finally, although institutional policy requires that interpreters are used for all communication with patients with LEP, we were unable to ascertain that this policy was adhered to in all of the calls in this study, and we could not ascertain the quality of interpretation in each encounter.

## Conclusions

In summary, among patients empaneled in a large primary care setting, IS callers who utilized a computer-aided, nurse-led telephone triage system were more likely to receive recommendations for higher acuity care visits compared to non-IS callers. IS callers were also less likely to follow the recommendation and had a higher rate of surrogate caller use. Additional research is needed to further understand why telephone recommendations to IS patients have more urgent endpoints and why the IS patients are less likely than non-IS patients to follow the triage recommendations. Finally, efforts to refine or tailor telephone triage to better serve patients with LEP seem warranted through quality improvement and research initiatives, such as use of video technology to augment objectivity in system assessment and communication.

## Abbreviations

AOR: Adjusted odds ratio
CCI: Charlson Comorbidity Index
ED: Emergency department
IS: Interpreter services
LEP: Limited English proficiency
OR: Odds ratio
